# α-Ketoglutarate Promotes Pancreatic Progenitor-Like Cell Proliferation

**DOI:** 10.3390/ijms19040943

**Published:** 2018-03-22

**Authors:** Jing Song, Dongshen Ma, Yun Xing, Shanshan Tang, Murad Alahdal, Jiamin Guo, Yi Pan, Yanfeng Zhang, Yumeng Shen, Qiong Wu, Zhou Lu, Liang Jin

**Affiliations:** State Key Laboratory of Natural Medicines, Jiangsu Key Laboratory of Drug Screening, School of Life Science and Technology, China Pharmaceutical University, 24 Tongjiaxiang, Nanjing 210009, China; livelypretty@163.com (J.S.); madongshen89@163.com (D.M.); xingyun_503@163.com (Y.X.); Sandycheekstang@163.com (S.T.); 17761713107@163.com (M.A.); 1622030924@stu.cpu.edu.cn (J.G.); 1020162543@cpu.edu.cn (Y.P.); yfzhang@cpu.edu.cn (Y.Z.); sym_penguins@163.com (Y.S.); WuQiong_92@163.com (Q.W.); zhoulusc@163.com (Z.L.)

**Keywords:** pancreatic progenitor-like cells, α-ketoglutarate, proliferation

## Abstract

A major source of β cell generation is pancreatic progenitor-like cell differentiation. Multiple studies have confirmed that stem cell metabolism plays important roles in self-renewal and proliferation. In the absence of glucose, glutamine provides the energy for cell division and growth. Furthermore, α-ketoglutarate (αKG), a precursor for glutamine synthesis, is sufficient for enabling glutamine-independent cell proliferation. We have demonstrated that αKG contributes to the large-scale proliferation of pancreatic progenitor-like cells that can provide an ample amount of clinically relevant β cells. We compared the mRNA expression of a subset of genes, the abundance of ATP, reactive oxide species, mitochondrial number, and the colony-forming frequency between mouse pancreatic CD133^+^ and CD133^−^ cells. We employed Real-Time PCR, immunostaining and passage assays to investigate self-renewal and proliferation of pancreatic progenitor-like cells in a 3D culture system in the presence and absence of αKG. The energy metabolism of CD133^+^ cells was more prone to oxidative phosphorylation. However, in the 3D culture system, when αKG was supplemented to the culture medium, the proliferation of the pancreatic progenitor-like cells was significantly elevated. We confirmed that the presence of αKG correlated with the up-regulation of Ten-Eleven Translocation (Tet). αKG can promote the proliferation of pancreatic progenitor-like cells via the up-regulation of Tet.

## 1. Introduction

**Figure A1:**
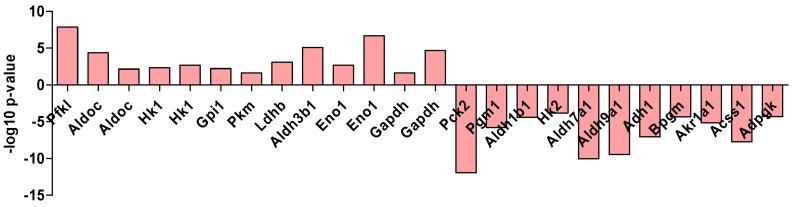
Significantly changed genes enriched in different KEGG pathways (represented by *p*-values) in progenitor-like cells cultured in vitro, compared to pancreatic single cells. Each column represents the mRNA level of the glycolysis-related enzymes. As shown, a portion of the glycolysis-related enzymes are up-regulated in progenitor-like cells.

**Figure A2:**
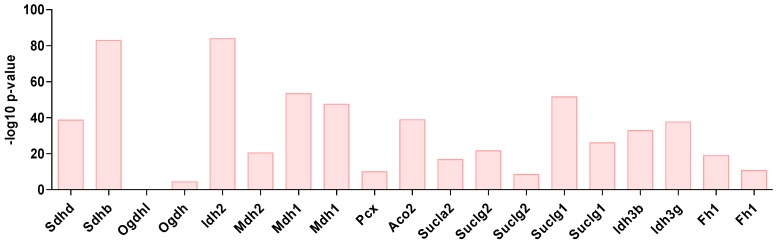
Significantly changed genes enriched in different KEGG pathways (represented by *p*-values) in progenitor-like cells cultured in vitro, compared to pancreatic single cells. Each column represents the mRNA level of the tricarboxylic acid cycle-related enzymes. As shown, all of the tricarboxylic acid cycle-related enzymes are up-regulated in progenitor-like cells.

Pancreatic islet transplantation has become a promising therapy for Type 1 diabetes (T1D) and Type 2 diabetes (T2D) for decades [1,2]. However, the shortage of donor islets hampers clinical application. Pancreatic progenitor-like cells that self-renew and differentiate into β cells can become a new source for β cell generation and help overcome the deficiency of donor islets [3,4]. The consensus is that pancreatic progenitor-like cells are located in the pancreas duct in vivo [5]. Many laboratories use CD133 as a surface marker for the identification of pancreatic ductal epithelial cells as it has the ability to self-renew and differentiate [6,7,8]. In our study, we employed the 3D culture system to propagate pancreatic progenitor-like cells derived from CD133^+^ cells in vitro. The metabolism of stem cells or progenitor cells plays crucial roles in self-renewal and proliferation [9,10]. However, there exists the possibility of metabolic differences between CD133^+^ and CD133^−^ cells. In fact, our previous results suggest that there are significant metabolic differences between CD133^+^ and CD133^−^ cells [11] (Figure A1 and Figure A2, Table A1).

CD133, CK7, and Sox9 are common markers for pancreatic ductal cells. Pdx1 (pancreatic and duodenal homeobox 1) is crucial for pancreatic development and a classic marker for pancreas specificity in the early pancreatic epithelium, permitting its proliferation, branching and subsequent differentiation. When cultured in vitro, pancreatic ductal-origin cell progenitors maintained a stable ductal epithelium phenotype and proliferated. Moreover, the progenitors expressed Pdx1 and differentiated into cell lines [5,6]. Cells expressing the endocrine precursors, Ngn3, Nkx6.1, Neurod1 and Pax4, will differentiate into endocrine cells. Insulin1 (ins1) and glucagon are markers of hormone-expressing endocrine cells, whereas Ki67 and CyclinD1 are proliferation related genes. Higher expression of these genes correlates with enhancement of cellular proliferation.

Tricarboxylic acid cycle (TCA) intermediates have effects on the energy metabolism of stem cells or progenitor cells [12,13]. α-ketoglutarate (αKG) is an important metabolic intermediate in the TCA cycle [14,15] and is dependent on cellular membrane transporters for its uptake. In vitro, supplementation of mouse embryonic stem cells (mESCs) with cell-permeable αKG directly supports self-renewal [16]. When dm-αKG, a chemical cousin of αKG, is added to the culture medium, enhanced cellular self-renewal is observed. However, dm-αKG also promotes histone and DNA demethylation, leading to the suppression and differentiation of naive-state mouse embryonic stem cells (mESCs) [17].

αKG, as a cofactor of Ten-Eleven Translocation (TET) proteins, can promote the expression of Tet genes. Tet activates DNA demethylation and can silence genes related to the differentiation of embryonic stem cells by keeping their pluripotency genes active. This regulation helps maintain the pluripotency of embryonic stem cells [18,19]. Mitochondrial synthase binds αKG and blocks its activity, suppressing the mTOR signaling pathway. Also, Tet has been reported to promote the proliferation of other stem cells [20,21]. However, there has been no report on the regulation of αKG on pancreatic progenitor-like cells. Pancreas progenitor-like cells derived from adult pancreatic duct have the capacity of self-renewal and differentiation. Features of the progenitor-like cells are similar to the pancreas progenitor cells (stem cells) which are the specialized cells in the early stage of the pancreas development and may have similar metabolic characteristics as compared to mESC. Here, we report the role of αKG on the proliferation and self-renewal of pancreatic progenitor-like cells in vitro.

## 2. Results

### 2.1. Metabolism Characterization of Pancreatic Progenitor-Like Cells from the Adult Mouse Pancreas

**Figure 1:**
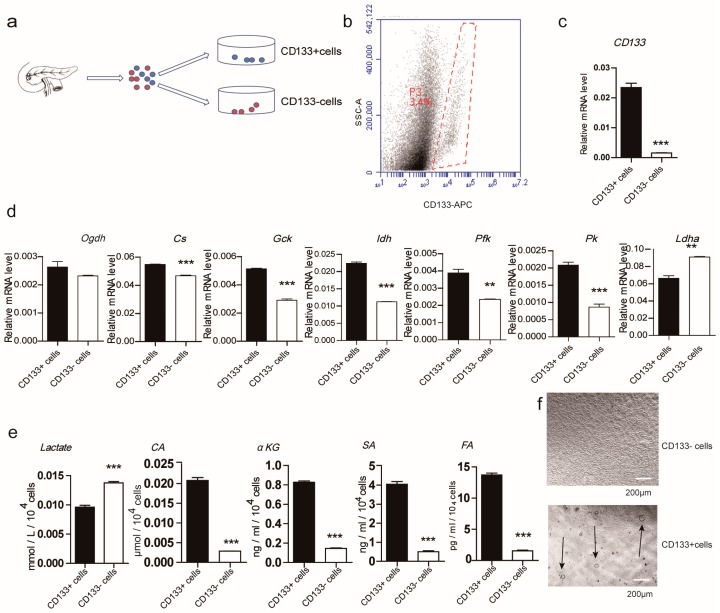
The metabolism levels of CD133^+^ and CD133^−^ cells in the adult murine pancreas. (**a**) CD133^+^ and CD133^−^ cells were freshly sorted by flow cytometry from pancreatic single cells; (**b**) the frequency of CD133^+^ cells in whole pancreatic cells was detected by flow cytometry; (**c**) the mRNA expression of CD133 and (**d**) indicated glucose metabolism related speed limit enzymes of freshly sorted CD133^+^ and CD133^−^ cells were evaluated by Real-time PCR; (**e**) glucose metabolism intermediates from CD133^+^/CD133^−^ cells were tested by analysis kits, in accordance with the manufacturers’ instructions; (**f**) the colony forming frequencies of CD133^+^ cells and CD133^−^ cells in the 3D culture system were counted under an inverted microscope. The arrows are used to point colonies for easy viewing and identification Results are shown as means + SEM and represent three independent experiments. *** *p* < 0.001 versus CD133^+^ cells; ** *p* < 0.01 versus CD133^+^ cells.

To test the metabolic differences between CD133^+^ cells and CD133^−^ cells in vivo, a freshly harvested adult mouse pancreas was first digested into single cells, and the CD133^+^ and CD133^−^ sub-populations were immediately sorted utilizing flowcytometry (Figure 1a). CD133^+^ cells accounted for 2–6% of the total pancreatic cells (Figure 1b). The expression of CD133 in the isolated CD133^+^ cell population was significantly higher than that in the CD133^−^ population, indicating that the harvested sub-population of CD133^+^ cells was relatively pure (Figure 1c). We compared the differences in metabolic pathways between the two populations immediately after sorting. The measured mRNA expressions of four glucose metabolism rate-limiting enzymes were higher in CD133^+^ cells than in CD133^−^ cells, except for α-ketoglutaric dehydrogenase (*Ogdh*) and lactate dehydrogenase (*Ldha*), which were lower. Compared to the CD133^−^ cells, the mRNA expression of the TCA cycle speed limit enzymes, citrate synthase (*Cs*) and isocitrate dehydrogenase (*Idh*), in CD133^+^ cells were significantly increased, but the mRNA expression of *Ogdh* was unchanged. Moreover, the *Ldha* mRNA expression in CD133^+^ cells was lower than in the CD133^−^ cells (Figure 1d). These results suggest that the energy metabolism of CD133^+^ cells is more dependent on oxidative phosphorylation, compared to that of CD133^−^ cells. Lastly, measurement of the glucose TCA metabolite intermediates was performed for both CD133^+^ and CD133^−^ cells. Citrate (CA), α-ketoglutarate (αKG), succinate (SA) and fumarate (FA) were found to be in a higher content in CD133^+^ cells, while glycolytic metabolites, such as lactate, had a higher content in CD133^−^ cells (Figure 1e). Moreover, the CD133^+^ cells exhibited high colony forming frequency, while CD133^−^ cells could not form ring colonies (Figure 1f). From these results, it appears that the CD133^+^ cells in the mouse pancreas are active rather than static.

**Figure 2:**
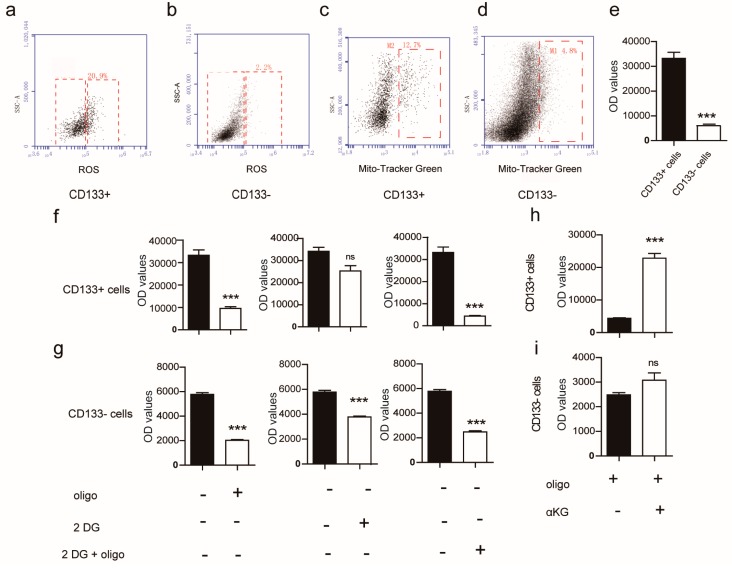
The analyses of mitochondria function in CD133^+^/CD133^−^ cells. (**a**,**b**) dot plot of reactive oxygen species (ROS) expression in CD133^+^/CD133^−^ cells was measured by flow cytometry; (**c**,**d**) the quantity of mitochondria in CD133^+^/CD133^−^ cells was detected by a Mito-tracker green staining kit in accordance with the manufacturer’s instructions; (**e**) the relative ATP level in CD133^+^ and CD133^−^ cells was tested by a ATP detection kit; (**f**,**g**) the CD133^+^/CD133^−^ cells were respectively treated with 2-DG (2 mM) or oligomycin (0.1 µg/mL), (**h**,**i**) and with αKG (8 mM) as indicated; the ATP level was tested by an ATP detection kit following the manufacturer’s instructions. Results are shown as means + SEM and represent three independent experiments. “+” indicates positive cells and ”−“indicates negative cells. *** *p* < 0.001 versus CD133^+^ cells; ns, no significant difference.

**Figure A3:**
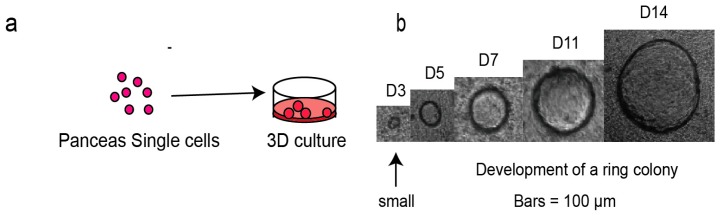

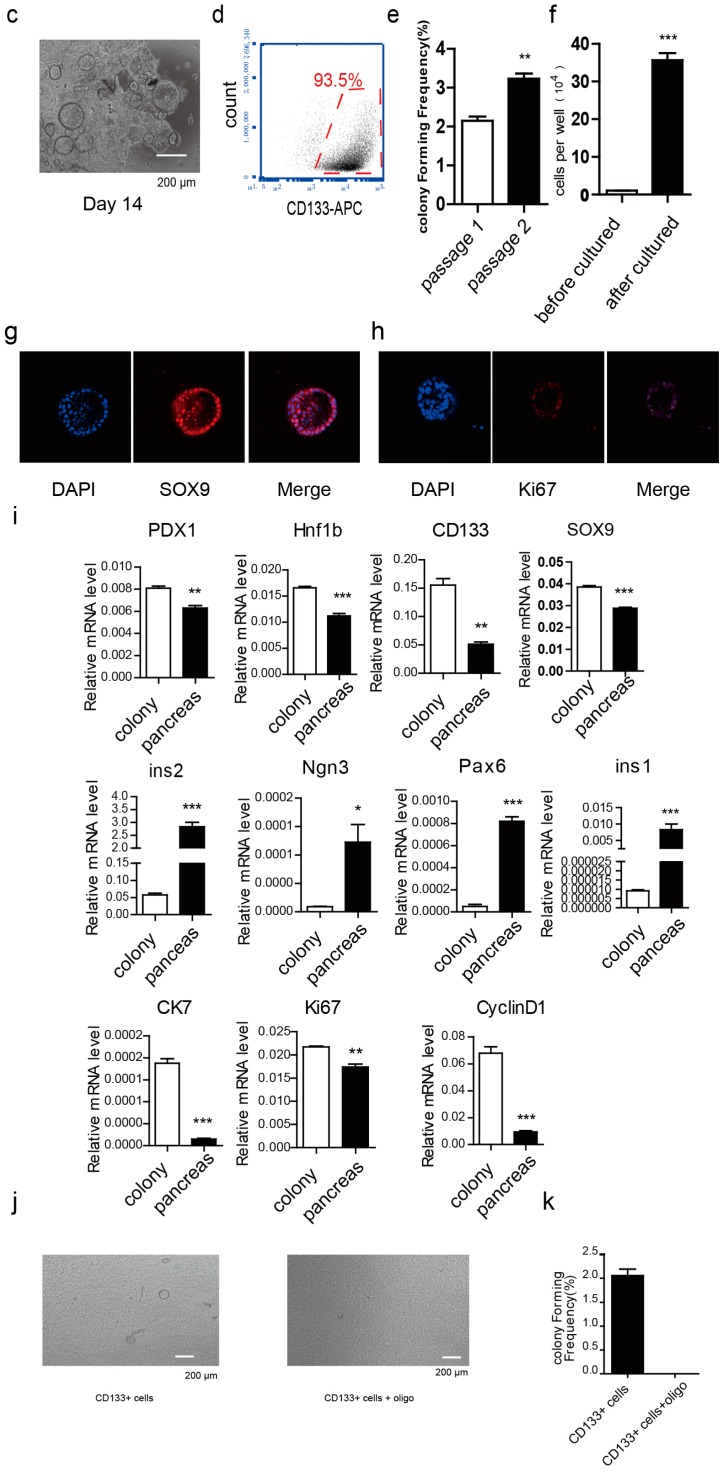
Application of the 3D culture system for the enrichment of CD133^+^ cells. (**a**) Schematic of ring colonies formed by pancreatic single cells; (**b**) representative photograph of the growth of a ring colony taken by inverted fluorescence microscope; (**c**) representative photograph of ring colonies on the 14th day taken by inverted fluorescence microscope; (**d**) the proportion of CD133^+^ cells in ring colonies; (**e**) colony forming frequencies of different passages; (**f**) the proliferation of pancreatic progenitor-like cells is reflected in the number of cells per well, counted with a cell counter; (**g**,**h**) CLSM images of a ring colony, which showed the expressions of Sox9 and Ki67 in the 3D culture system. Nuclei were stained with DAPI; (**i**) indicates the progenitor-related genes, Sox9, Pdx1, Hnf1b and CD133; differentiation-related genes, ins1, ins2, ngn3 and Pax6; ductal epithelial cells-related genes; CK7; and proliferation-related genes, CyclinD1 and Ki67 in colonies and pancreas; (**j**) photographs taken by inverted fluorescence microscope of CD133^+^ cells or oligo-treated CD133^+^ cells; (**k**) colony forming frequencies of CD133^+^ cells and oligo-treated CD133^+^ cells, defined by the number of colonies/the number of plated cells per well. “Relative mRNA levels” are the measured mRNA levels compared to the mRNA levels of the internal standard, cyclophilin A. *** *p* < 0.001 versus 1.0 g/L colony group; ** *p* < 0.01 versus 1.0 g/L colony group. * *p* < 0.05 versus 1.0 g/L colony group. Results represent three independent experiments.

Reactive Oxygen Species (ROS) will increase when oxidative phosphorylation is high. Therefore, ROS levels were used as an indicator for measuring oxidative phosphorylation in the two cellular isolated sub-populations. We found that the ROS level in CD133^+^ cells was significantly higher compared to that of CD133^−^ cells, indicating that energy metabolism of CD133^+^ cells was more prone to oxidative phosphorylation compared to the CD133^−^ cells (Figure 2a,b). Next, we evaluated the number of mitochondria between the two populations and found that CD133^+^ cells had significantly more mitochondria than CD133^−^ cells (Figure 2c,d). The mitochondrial number will increase to meet the energy demand in the cells that have a high metabolic rate [22]. We concluded that the CD133^+^ cells isolated from the mouse pancreas were more metabolically active than the CD133^−^ cells. The fact that CD133^+^ cells were more metabolically active supports the notion that these cells are more prone to oxidative phosphorylation. Next, we measured the ATP levels and found that the CD133^+^ cells contained higher amounts of ATP, reflecting a higher metabolic rate, compared to that of the CD133^−^ cells, which contained lower amounts of ATP (Figure 2e). We then assayed the cell populations in the presence and absence of 2-Deoxy-d-Glucose (2-DG) and oligomycin, respectively to further investigate the metabolic pathways of the two sub-populations of cells. 2-DG is a competitive inhibitor of glucose and subsequent glycolytic inhibitor. 2-DG generates 6-phosphoric-acid-2-deoxy-d-glucose, which cannot be converted into 6-phosphoric-acid-fructose by phosphate-glucose-isomerase. Thus, it inhibits the subsequent steps of glycolysis. Oligomycin is an inhibitor of oxidative phosphorylation in mammalian cells. It effectively binds the functional subunit, F0, of mitochondrial F0F1ATP synthase to change the conformation of ATP synthase, thereby inhibiting the proton flux in the mitochondrial membrane gap from flowing back to the mitochondrial matrix. As a result, the synthesis of ATP is blocked and results in the lack of energy required for metabolism. CD133^+^ cells grown in the presence of 2-DG did not affect the energy metabolism, while the presence of oligomycin had a significant effect on the energy metabolism of CD133^+^ cells, suggesting that the energy flux of CD133^+^ cells primarily depends on oxidative phosphorylation. Moreover, both 2-DG and oligomycin had significant effects on the energy metabolism of CD133^−^ cells, indicating that the energy metabolism of CD133^−^ cells depends on both oxidative phosphorylation and glycolysis (Figure 2f,g). Next, we grew CD133^+^ cells in the presence of the mitochondrial ATP synthase inhibitor, oligomycin, to perturb the proliferation of the cells in 3D culture. We observed that CD133^+^ cells could not form colonies when grown in the presence of oligomycin (Figure A3j,k), suggesting that oxidative phosphorylation plays an important role in CD133^+^ cells’ metabolism.

Taken together, these data suggest that the CD133^+^ cells isolated from the mouse pancreas have a higher metabolic rate and they are more prone to oxidative phosphorylation compared to those of the CD133^−^ cells.

### 2.2. αKG Promoted the Proliferation of Pancreatic Progenitor-Like Cells

**Figure 3:**
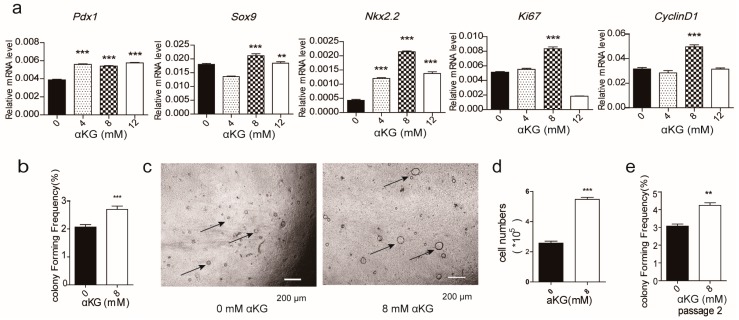

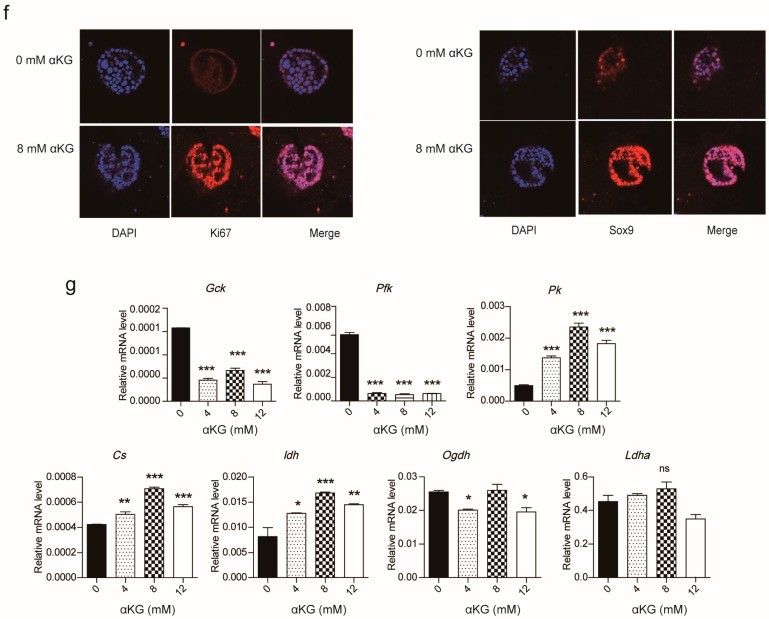
α-ketoglutarate (αKG) affected the proliferation of pancreatic progenitor-like cells in 3D culture. (**a**) The pancreatic single cells were cultured in a 3D cultured system with αKG at the indicated doses; the progenitor- and proliferation-related mRNAs were detected by Real-time PCR; (**b**) 0 mM and 8 mM αKG was added into the 3D culture system at the culture’s beginning; the colony forming frequency was evaluated via the described method, and (**c**) the colonies were observed under an inverted microscope; (**d**) single cells/well digested from αKG cultured colonies were counted; (**e**) the self-renewal ability in 3D culture with 0 mM αKG and 8 mM αKG; (**f**) the Sox9 and Ki67 levels in colonies were detected by immunofluorescence under laser scanning confocal microscopy; nuclei were stained with DAPI; (**g**) The mRNA levels of glucose metabolism-related speed limit enzymes of αKG treated colonies were tested by Real-time PCR. The arrows are used to point colonies for easy viewing and identification. Results are shown as mean + SEM and represent three independent experiments. *** *p* < 0.001 versus 0 mM αKG group; ** *p* < 0.01 versus 0 mM αKG group; * *p* < 0.05 versus 0 mM αKG group; ns, no significant difference.

The ATP levels of the two cell populations were measured after each cell type was incubated in the presence or absence of oligomycin and αKG, respectively. Consequently, we found that the ATP level of CD133^+^ cells had increased when incubated in the presence of αKG; however, the ATP level of CD133^−^ cells was unchanged under similar conditions. This confirmed that αKG affects the regulation of substrate phosphorylation in CD133^+^ cells (Figure 2h,i). Subsequently, αKG was introduced to the 3D culture system. Surprisingly, the expression of progenitor-related genes, *Pdx1*, *Sox9*, and *Nkx2.2*, increased significantly and the proliferation markers, *CyclinD1* and *Ki67*, were also up-regulated in the presence of αKG (8 mM) (Figure 3a). As expected, the colonies with αKG (8 mM) exhibited a higher colony forming frequency (derived from 10,000 cells) with significantly more large-sized colonies (Figure 3b,c). However, colonies grown in the presence of 12 mM αKG demonstrated a decrease in proliferation, possibly because αKG caused the pH of the medium to decrease, having a detrimental effect on cell growth (data not shown). We also demonstrated that both pancreatic progenitor-like-cell proliferation (presented by cell numbers per well) and self-renewal capacity, grown in the presence of 8 mM αKG, increased (Figure 3d,e). Next, we examined the expression of Ki67 by immunofluorescence and found that it was up-regulated. In fact, the majority of the cells were Ki67+ cells when grown in the presence of 8 mM αKG. Also, the expression of the pancreatic progenitor-related gene, Sox9, was increased (Figure 3f). Furthermore, we found that the gene expression of the tricarboxylic acid cycle rate-limiting enzymes was increased, indicative that αKG plays a regulatory role in the metabolism of the pancreatic progenitor-like cells (Figure 3g).

These data demonstrate that αKG can promote the energy metabolism of Pdx1+/Sox9+ pancreatic progenitor-like colonies via the tricarboxylic acid cycle. More importantly, αKG can specifically promote the proliferation of Pdx1+/Sox9+ pancreatic progenitor-like colonies, increase the colony forming frequency of pancreatic progenitor-like colonies in vitro, and improve the transcription of progenitor-related genes.

### 2.3. Succinate Suppresses the Proliferation of Pancreatic Progenitor-Like Cells

**Figure 4:**
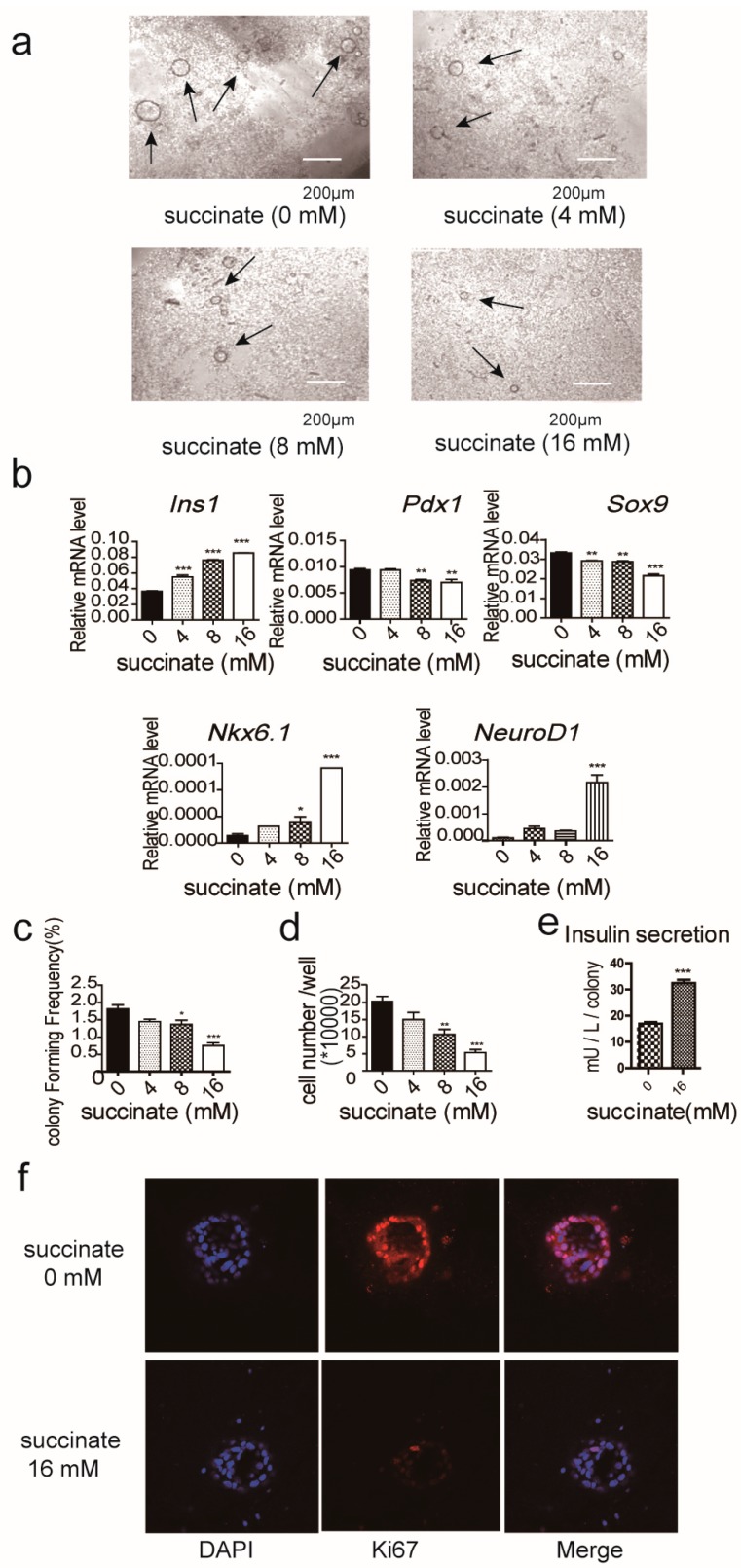
The effect of succinate on the proliferation of pancreatic progenitor-like cells in 3D culture. (**a**) The digested pancreatic single cells were cultured with the addition of 0 mM, 4 mM, 8 mM or 16 mM succinate for 14 days; the colonies were observed under an inverted microscope; nuclei were stained with DAPI; (**b**) the expressions of progenitor- and differentiation-related genes were analyzed by Real-time PCR; (**c**) tThe colony forming frequency was calculated as described; (**d**) the cell numbers/wells from colonies were counted by utilizing a cell counter; (**e**) the insulin secretion of colonies was detected by an insulin detection ELISA kit, in accordance with the manufacturer’s instructions; (**f**) the Ki67 levels in the 3D culture with 0 mM and 16 mM succinate were detected by immunofluorescence under a laser scanning confocal microscopy; nuclei were stained with DAPI. The arrows are used to point colonies for easy viewing and identification. Results are shown as means + SEM and represent three independent experiments. *** *p* < 0.001 versus 0 mM succinate group; ** *p* < 0.01 versus 0 mM succinate group; * *p* < 0.05 versus 0 mM succinate group.

Succinate, an αKG-dependent dioxygenase competitive inhibitor promotes differentiation of mESC [23]. Therefore, we examined its effect on pancreatic progenitor-like cell growth, via the presence and absence of 4, 12 and 16 mM succinate, respectively. We observed that the diameter of the colonies grown in the presence of 4 mM succinate was larger than the colonies grown in the presence of 12 mM succinate (Figure 4a). We observed that the gene expression of pluripotency markers, *Pdx1* and *Sox9*, were decreased, while gene expression of the differentiation markers, *insulin1*, *Nkx6.1* and *Neurod1*, were increased in cells grown in the presence of 4, 12 and 16 mM succinate, respectively (Figure 4b). These results suggest that succinate can promote the mRNA expression of genes related to cellular differentiation. However, the addition of cell permeable succinate resulted in a decreased colony forming frequency and in decreased cell numbers per well at all concentrations of succinate measured (Figure 4c,d). We performed an insulin secretion assay and KI67 staining and found that the insulin secretion ability of the colonies cultured in the presence of succinate was increased (Figure 4e), while the expression of KI67 was decreased (Figure 4f). These results show that inhibition of αKG metabolism suppressed the proliferation of pancreatic progenitor-like cells and promoted the expression of genes related to cellular differentiation.

### 2.4. Regulation Proliferation of Pancreatic Progenitor-Like Cells via Tet

**Figure 5:**
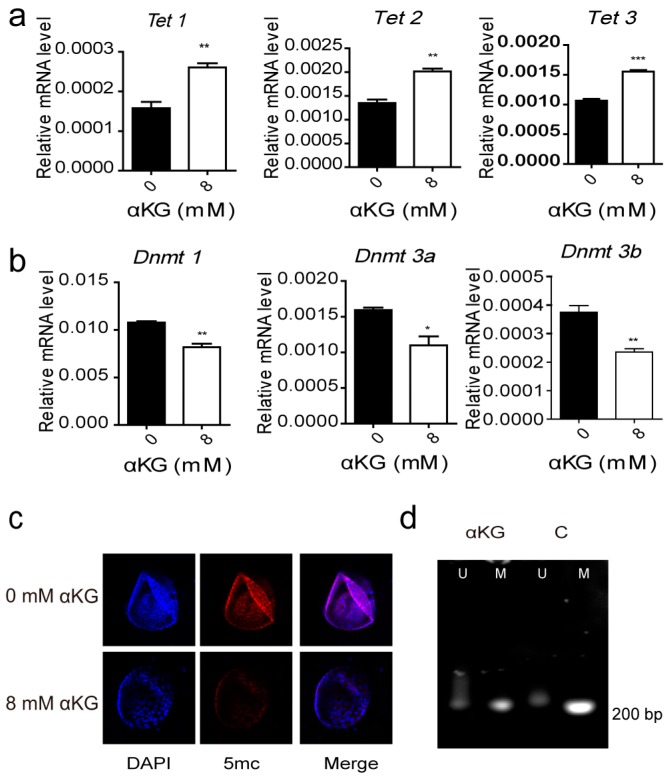
The detection of DNA demethylation levels in αKG treated colonies. (**a**) the expression of Tet and (**b**) Dnmt tested by Real-time PCR in the 3D cultured colonies containing 0 mM and 8 mM αKG; (**c**) the expression of 5mc in the 3D cultures with 0 mM and 8 mM αKG was observed by immunofluorescence under a laser scanning confocal microscopy; nuclei were stained with DAPI; (**d**) methylation-specific PCR (MSP) analysis of the CyclinD1 gene promoter in the 3D culture with or without αKG by PCR (U = unmethylated DNA, M = methylated DNA). Results are shown as means + SEM and represent three independent experiments. *** *p* < 0.001 versus 0 mM αKG group; ** *p* < 0.01 versus 0 mM αKG group; * *p* < 0.05 versus 0 mM αKG group.

Tet is an αKG and Fe^2+^ dependent dioxygenase, and it can contribute to the regulation of progenitor-related gene expression. The mRNA expressions of Tet1, Tet2 and Tet3 were increased in the pancreatic progenitor-like cells cultured in the presence of αKG (Figure 5a). Meanwhile, the mRNA expression of DNA methyltransferase (DNMT) was decreased (Figure 5b). To assess the effect of the Tet enzymes on the pancreatic progenitor-like cells, we measured the level of 5-methylcytosine (5mc), utilizing an immunofluorescence assay (Figure 5c) and found that the expression of 5mc was down-regulated in the pancreatic progenitor-like cells cultured in the presence of αKG. To further investigate whether the pancreatic progenitor-like cells proliferation was associated with demethylation of the proliferation-related gene promoters, like CyclinD1, we performed a methylation-specific PCR (MSP) analysis on the colonies grown in the 3D culture in the presence or absence of αKG. We found that demethylation of the CyclinD1 gene promoter occurred in colonies cultured in the presence of αKG (Figure 5d). These data suggest a significant role of Tet enzymes in αKG-promoted proliferation of pancreatic progenitor-like cells.

## 3. Discussion

The system of choice for the proliferation of pancreatic cells was 3D culturing medium, as a growing body of evidence has suggested that 3D cell culture systems, in contrast to the 2D culture system, represent the actual microenvironment where cells reside in tissues more accurately. Thus, the behavior of 3D-cultured cells is more reflective of in vivo cellular responses. As anticipated, ring colonies grown in a 3D culture system have the ability to self-renew and proliferate. Thus, a 3D culture system is suitable for the enrichment of CD133^+^ cells (Figure A3a–i).

Pancreatic islet transplantation has become a promising therapy for type 1 and late stage type 2 diabetes for decades. However, the shortage of donor islets hampers clinical application. As pancreatic progenitor-like cells can self-renew and differentiate into β cells, pancreatic progenitor-like cells have the potential to become a new source for β cell generation in the clinical setting. Many studies have confirmed that the metabolism of stem cells plays an important role in self-renewal and proliferation. CD133 has been proposed to act as an organizer of cell membrane topology. Therefore, the possibility exists that there may be metabolic differences between CD133^+^ and CD133^−^ cells. Glutamine is known to be an important energy source for cell growth, and αKG, which is a precursor for glutamine synthesis, can also provide energy for cell growth. Our study examined whether αKG can sufficiently promote proliferation of pancreatic progenitor-like cells and provide ample β cells that are clinically relevant for the treatment of type I and II diabetes.

In general, adult progenitor cells are normally quiescent with only a small number of cells that differentiate to meet the demand for normal physiological function [24,25,26]. Our results demonstrated that the metabolic energy rate of CD133^+^ cells is higher than that of the CD133^−^ cells. The observed increased metabolic energy rate of CD133^+^ cells is quite different from normal progenitor cell metabolism. As we know, progenitor cells can exist in various metastable states, such as the “naïve or primed” state [27,28]. There is a possibility that the pancreatic progenitor-like cells are in the primed state. However, why pancreatic progenitor-like cells would be in the primed state is still unknown.

In different types of stem cells, glucose metabolism has been shown to be important for differentiation, survival [29,30] and proliferation [31]. Thus, an increased expression of glucose metabolizing rate-limiting enzymes is required for cell proliferation and is sufficient to increase the cellular lifespan [32], no matter whether the glycolytic or the TCA cycle is the important energy source for the metabolism of stem cells [33,34]. For example, both stimulation of glycolysis in pluripotent stem cells and inhibition of mitochondrial respiration could influence the stem characteristics of a cell [31,35]. In CD133^+^ cells, not only the expression of the TCA-related enzymes, but also ATP levels, were higher than that in CD133^−^ cells which may be a prerequisite for the proliferation of CD133^+^ cells.

This study revealed that αKG could promote the self-renewal and proliferation of pancreatic progenitor-like cells through TET-mediated DNA demethylation.

There is a catalytic structure domain (Catalytic-dioxygenase domain) near the c-terminal region of Tet protein. The structure of the domain has three metal ions (Fe^2+^) and one αKG binding site, and upstream of the catalytic domain structure, exists a Cys-rich domain. The structure of the Tet protein catalytic knot domain and the Cys-rich domain demonstrated αKG and Fe^2+^ dependent oxygen^+2^ enzyme activities [36,37]. Together with αKG and Fe^2+^, Tet protein participated in DNA demethylation through the oxidation of 5mc to 5hmc [38,39]. In mESC, when the expression of Tet1 was restrained, the 5mc/5hmc totipotency factor and nanog promoter was increased, but its expression was decreased. In pancreatic progenitor-like cells, not only the expression of Tet but also many pluripotent genes, like *Pdx1*, *Sox9*, *Nkx2.2*, and the proliferation-related gene, *CyclinD1*, were increased. We also revealed that the promoter of the CyclinD1 had been demethylated, suggesting that Tet had positive effects on targeted gene expression [21] and Tet promoted the proliferation of pancreatic progenitor-like cells through demethylation of the CyclinD1 promoter.

In our studies, the expression of DNA methyltransferase (DNMT) was decreased when treated with αKG. There are two reasons for this—one reason is that Tet1 strongly binds to unmethylated-CPG (Nucleotide pair) rich regions via its CXXC domain which limits the accessibility of DNMTs. The other reason is that Tet1 can convert 5mc to 5hmc and may further limit the binding of DNMTs. Furthermore, Tet could oxidize 5mc [40,41]. The increased demethylation of Tet and the decreased methylation of DNMT can reduce the methylation level of the entire genome [42]. In mESCs, the absence of DNA methyltransferases, Dnmt1, Dnmt3a and Dnmt3b, could maintain the self-renewal of mESCs [43,44], which is consistent with our findings.

αKG is an intermediate of the tricarboxylic acid cycle and has an important influence on the energy metabolism of mitochondria, and the energy metabolism of mitochondria has a big effect on the energy state of stem cells. Bryce W. Carey showed that naïve ESCs exhibit elevated αKG/succinate ratios that promote histone/DNA demethylation and maintain pluripotency. Direct manipulation of the intracellular αKG/succinate ratio is sufficient to regulate multiple chromatin modifications, including H3K27me3 and Ten eleven translocation (Tet)-dependent DNA demethylation that contribute to the regulation of pluripotency-associated gene expression. In vitro, supplementation with cell-permeable αKG directly supports ESC self-renewal, while cell-permeable succinate promotes differentiation [16]. Furthermore, αKG can also affect the growth and metabolism of other cells, such as Pancreatic Ductal Adenocarcinoma (PDAC). Ju-Won Seo showed that Pancreatic Ductal Adenocarcinoma (PDAC) cells require both autophagy and typical glutamine transporters to maintain intracellular glutamine levels. Moreover, autophagy inhibition and glutamine deprivation did not induce cell death, while glutamine deprivation dramatically activated apoptotic cell death upon autophagy inhibition. Interestingly, the addition of α-ketoglutarate significantly rescued the apoptotic cell death caused by the combination of the inhibition of autophagy with glutamine [45].

Generally speaking, adult stem cells are a relatively inactive group of cells in vivo, and the mitochondria are less active. However, in pancreatic progenitor-like cells, the relative ATP and the mitochondria number of CD133^+^ cells were significantly greater than in CD133^−^ cells. Therefore, mitochondrial energy metabolism affects the state of stem cells. In our study, it was shown that αKG can affect CD133^+^ cells’ energy metabolism. However, further studies are needed to determine whether αKG can maintain the pluripotency of pancreatic progenitor-like cells.

## 4. Materials and Methods

### 4.1. Animals

Normal C57BL/6J mice (6–8 weeks old) were bought from Animal Research Center of Yang Zhou University (Nanjing, China). All the care of animals and treatments were accorded to international laws and policies (EEC Council Directive 86/609, 1987) and complied with the regulations of the animal ethics committee of China Pharmaceutical University (approval No. CPUSPF/SQ-16025, 12 February 2015).

### 4.2. Single Cell Preparation and 3D Culturing

The pancreas was first isolated from a 6–8-week-old C57BL/6J mouse and cut into 1 mm^3^ pieces on a 100 mm^2^ cold dish in 3 min. Then, it was transferred into a 15 mL centrifuge tube and digested with 10 mg/mL collagenase II and 150 U/mL deoxyribonuclease I. After that, the mixture was transferred to a centrifuge tube and incubated in a 37 °C water-bath for 7 min. The digestive juice was mixed gently by syringe 15 times and then transferred to a 37 °C water bath for an additional 10 min. The digestive juice was mixed by syringe 15 times, once again. Finally, the centrifuge tube was put in a 37 °C water bath for 5 min, and the digestive juice was mixed by syringe 5 times. Single cells were obtained, as determined by a phase-contrast microscope. Live cells were counted and seeded into a 24-well ultra-low attachment plate (Corning, NY, USA) in 3D culture medium (10,000 cells/well). Also, CD133^+^ cells and CD133^−^ cells sorted from the pancreas by flowcytometry (BD FACS Aria, New York, NY, USA) were cultured under the same conditions. The 3D culture medium consisted of 10% Matrigel (Corning), 1% methylcellulose (Sigma, St. Louis, MO, USA), 20 ng/mL EGF (Sigma), 10 mM Nicotinamide (Sigma), 2% B27 (Invitrogen, Carlsbad, CA, USA), 7.7% FBS (Gibco, California, USA), 100 U/mL penicillin, 100 μg/mL streptomycin (Gibco) and 5 μm SB 431542 (Sigma) in high glucose medium. Cells were cultured at 37 °C with 5% CO_2_ for 2 weeks with the medium left unchanged. During this time, “ring” colonies formed spontaneously from single cells. According to different experimental requirements, 2 μg of the TCA inhibitor, oligomycin A (Oligo, Sigma, St. Louis, MO, USA), with or without αKG, and a panel of concentrations of αKG or succinate were added into the culture medium prior to incubation. The number of colonies were counted under an inverted microscope (Olympus, Tokyo, Japan). The colony forming frequency is defined by the number of colonies per well/the number of plated cells per well. Single cells from the colonies were acquired through digestion in the presence of 0.25% trypsin for 5 min and were counted with a cell counter.

### 4.3. RNA Extraction

RNA extraction was performed following a standard procedure. Firstly, ring colonies in a 3D culture system were hand-picked and collected and transferred into a 1.5 mL tube. Secondly, both ring colonies and cells sorted from the pancreas were put separately into QiAzol Lysis Reagent (Qiagen, Hilden, Germany) to obtain cell lysate. Lastly, all samples were homogenized and RNA was extracted using a Qiagen RNeasy Mini kit (Qiagen).

### 4.4. Real-Time PCR and RT-PCR

Either Real-time PCR or RT-PCR was performed following a standard protocol. cDNA was prepared using 5 × All-In-One RT MasterMix (ABM, Richmond, BC, Canada) and RT-PCR was performed on a Thermo-Scientific PCR instrument. The RT-PCR products were separated and detected by running the samples on 1.5% agarose gel (Lonza, Basel, Switzerland). Real-time PCR was performed using EvaGreen 2 × qPCR MasterMix on Light Cycler 480 II (Roche, Basel, Switzerland). CyclophilinA was used as an internal reference. “Relative mRNA level” means the mRNA level relative to the cyclophilinA used as the internal reference. The primers used in RT-PCR and Real-time PCR are listed in Table 1.

### 4.5. Self-Renewal Assay

Colonies were cultured in 3D culture for 2 weeks and were hand-picked and counted under an inverted microscope. Single cells were acquired by digestion vialayering a 0.25% trypsin solution over the cells for 5 min. To test self-renewal ability, the isolated single cells from the colonies were counted and re-plated under the same conditions (10,000 cells per well). Two weeks later, the number of secondary colonies were trypsinized and counted.

### 4.6. Immunostaining

Ring colonies cultured in the presence or absence of α-ketoglutarate were harvested and fixed in 4% paraformaldehyde overnight at 4 °C. The next day, the cells were permeabilized with a 0.2% Triton-100 solution at room temperature (25 °C). Next, they were blocked with blocking buffer (Abcam, Cambridge, UK) at 4 °C overnight. Subsequently, the cells were incubated in the presence of the primary antibody at 4 °C overnight. The following day, the secondary antibody was added to the ring colonies, which were incubated at 37 °C. The final step involved adding DAPI to the cell solution to label the cellular DNA. All primary antibodies were from Abcam. The dilution of each antibody was as follows: Ki67 (1:200), Sox9 (1:200), CD133 (1:200). After treatment, photographs were taken by laser scanning confocal microscopy (Olympus, Tokyo, Japan) (CLSM) or inverted fluorescence microscope (Olympus).

### 4.7. Flow Cytometry and Cell Sorting

The ring colonies were hand-picked and digested in a solution of 0.25% trypsin. The cells were then stained with CD133-APC antibody (eBioscience, Hangzhou, China) at 1 μL/106 cells for 1 h on ice. The cells were washed twice in PBS, and the CD133^+^ cells were detected by Accuri C6 (BD). The pancreatic single cells were stained with CD133-APC antibody at 1 μL/106 cells for 1 h on ice. The cells were washed twice in PBS; the CD133^+^ cells were analyzed by Accuri C6 (BD) or sorted by Aria (BD).

### 4.8. ATP Level Determination

The ATP assays were performed using the ATP mini kit (Promega, Madison, WI, USA). Approximately 40,000,000 pancreatic single cells were stained with CD133-APC antibody, and then the CD133^+^ population and the CD133^−^ population were sorted with the flow cell sorter. We incubated the two sub-populations (10,000 cells per well) in a 96-cell culture plate in the presence of the glycolytic inhibitor, 2-Deoxy-d-glucose (2-DG) (Yuanye Biology, Shanghai, China) and the TCA inhibitor, oligomycin A (oligo) (Sigma), was added to the medium, simultaneously or separately, and incubated at 37 °C, 5% CO_2_, for 3 h. Then, the 96-well plate was brought to room temperature (25 °C). An ATP working solution was added to each well, and the chemical luminescence was acquired by a microplate reader. The relative chemical luminescence is representative of the ATP level.

### 4.9. Level of Reactive Oxide Species (ROS)

We performed the ROS analysis using a DCFH-DA kit (Sigma, St. Louis, MO, USA), following standard procedure. DCFH-DA has no fluorescence and can pass freely through the cell membrane. After it enters the cells, it becomes metabolized to DCFH which cannot pass the cell membrane. The ROS in the cells can oxidize DCFH. DCF has fluorescence that is detectable and is an indicator of ROS levels. The pancreatic single cells were first suspended in the DCFH-DA working solution at about 10^6^ cells/mL and incubated in a 37 °C water bath for 20 min. During the incubation, the solution was mixed every 3–5 min. Subsequently, the cells were washed three times in the working solution, and then serum-free medium was added to stop the reaction. The fluorescence of the cells was detected by Accuri C6 (BD). The fluorescence intensity represents the relative level of ROS.

### 4.10. Quantification of Mitochondrial Number

The mitochondrial quantification experiment was performed using a Mito-Tracker green kit (Biyuntian, Nanjing, China), following a standard procedure. The pancreatic single cells were first suspended with dye buffer at 10^6^ cells/mL and incubated at 37 °C for about 30 min. Then, Mito-Tracker Green Dyeing buffer was removed by centrifugation at 500× *g* for 5 min. The cells were washed twice with PBS, and the cells were resuspended in fresh culture medium at 37 °C. Then the cell fluorescence was detected by Accuri C6 (BD). The fluorescence intensity represents the relative mitochondrial number.

### 4.11. Intermediate Metabolite Assay

Intermediate metabolite experiments were performed using the lactate test kit (Jiancheng, Nanjing, China), CA ELISA kit (JiangLai, Shanghai, China), αKG ELISA kit (JiangLai, Shanghai, China), SA ELISA kit (JiangLai, Shanghai, China), and the FA ELISA kit (JiangLai, Shanghai, China), following a standard procedure. The two sub-populations of cells obtained from the pancreas homogenate were added with PBS. Next, a freeze-thaw cycle was performed three times, followed by centrifugation of the cells at 12,000 rpm for 20 min. The supernatant was decanted and saved. Lastly, the intermediate metabolite assay was performed with the kits mentioned previously. After, the chemical luminescence was acquired from the supernatant with a microplate reader; the results were recorded following the instructions.

### 4.12. Insulin Secretion Assay

The colonies cultured in the presence or absence of succinate were picked and cultured in 1640 medium (Gibco), supplemented with 0.2% FBS and 100 ng/mL activin A (Sigma) for 1–3 days. After the given incubation time the culture medium was replaced with a new medium that consisted of 1640, 500 nM PdBU (Sigma), 100 ng/mL FGF10 (Sigma) and 2% FBS, and the colonies were cultured during days 4–8; The culture medium was then exchanged with a third medium, supplemented with H-DMEM (Sigma), 1% B27, 50 ng/mL Noggin, 2 μM RA and 0.25 μM Sant1, in which colonies were cultured during days 9–14. Next, the culture medium was exchanged with a fourth medium supplemented with H-DMEM, 1% B27, and 1 μM Compund-E, and the colonies were cultured during days 14–18. After the final incubation, the colonies were placed into KRBE, consisting of 10% FBS for 12 h. The final culture medium was 100 µL KRBE, supplemented with 0.2% FBS and 16.7 mM theophylline, and colonies were incubated in this medium for 2 h at 37 °C. The insulin secreted from the colonies was in the medium, followed by a direct ELISA test utilizing an insulin kit (JiangLai), following a standard procedure.

### 4.13. Methylation-Specific PCR (MSP) Assay

Genomic DNA was obtained from colonies using a TIANamp Genomic DNA kit (TIANGEN, Beijing, China) following a standard procedure. The DNA obtained was treated with a DNA Bisulfite Conversion Kit (TIANGEN). Primers were designed, and the Methylation-specific PCR was performed using a Methylation-specific PCR (MSP) Kit (TIANGEN). The RT-PCR products were separated and detected by using 1.5% agarose (Lonza). Primers are listed in Table 1.

### 4.14. Statistical Analysis

Data are presented as means ± SEM. Two-tailed *t*-tests were used to assess the differences between experimental groups. One-way ANOVAs were used for multiple groups. Statistical significance was defined as * *p* < 0.05, ** *p* < 0.01 and *** *p* < 0.005.

## 5. Conclusions

In summary, our study confirmed that CD133^+^ cells are active and have a significantly higher metabolic rate compared to CD133^−^ cells. Moreover, αKG can promote pancreatic progenitor-like cells’ proliferation via the up-regulation of Tet. This approach has the potential to allow ample β cells to be acquired for pancreatic islet transplantation in a clinical setting. 

## Figures and Tables

**Table 1:** Primers used in experiments.

	Primer Name	Sequence (5′ to 3′)
1	CD133-F	CTCCCATCAGTGGATAGAGAACT
2	CD133-R	ATACCCCCTTTTGACGAGGCT
3	CK7-F	ACGGATGGGGCTAACTTACAA
4	CK7-R	AGTCCTCGATTTGCTCGAACT
5	CyclinD1-F	GCGTACCCTGACACCAATCTC
6	CyclinD1-R	CTCCTCTTCGCACTTCTGCTC
7	glucagon-F	TTACTTTGTGGCTGGATTGCTT
8	glucagon-R	AGTGGCGTTTGTCTTCATTCA
9	insulin1-F	CACTTCCTACCCCTGCTGG
10	insulin1-R	ACCACAAAGATGCTGTTTGACA
11	Nkx2.2-F	CCGGGCGGAGAAAGGTATG
12	Nkx2.2-R	CTGTAGGCGGAAAAGGGGA
13	Nkx6.1-F	CTGCACAGTATGGCCGAGATG
14	Nkx6.1-R	CCGGGTTATGTGAGCCCAA
15	Neurod1-F	ATGACCAAATCATACAGCGAGAG
16	Neurod1-R	TCTGCCTCGTGTTCCTCGT
17	Pdx1-F	CCCCAGTTTACAAGCTCGCT
18	Pdx1-R	CTCGGTTCCATTCGGGAAAGG
19	Sox9-F	GAGCCGGATCTGAAGAGGGA
20	Sox9-R	GCTTGACGTGTGGCTTGTTC
21	ngn3-F	AGTGCTCAGTTCCAATTCCAC
22	ngn3-R	CGGCTTCTTCGCTTTTTGCTG
23	Ki67-F	ATCATTGACCGCTCCTTTAGGT
24	Ki67-R	GCTCGCCTTGATGGTTCCT
25	Pax4-F	AGGGGGACTCTTTGTGAATGG
26	Pax4-R	ACCTGTGCGGTAGTAGCGT
27	Gck-F	TGAGCCGGATGCAGAAGGA
28	Gck-R	GCAACATCTTTACACTGGCCT
29	Pfk-F	GGAGGCGAGAACATCAAGCC
30	Pfk-R	CGGCCTTCCCTCGTAGTGA
31	Pk-F	GCCGCCTGGACATTGACTC
32	Pk-R	CCATGAGAGAAATTCAGCCGAG
33	Ogdh-F	GTTTCTTCAAACGTGGGGTTCT
34	Ogdh-R	GCATGATTCCAGGGGTCTCAAA
35	Cs-F	GGACAATTTTCCAACCAATCTGC
36	Cs-R	TCGGTTCATTCCCTCTGCATA
37	Idh-1-F	ATGCAAGGAGATGAAATGACACG
38	Idh-1-R	GCATCACGATTCTCTATGCCTAA
39	Ldha-F	CCGTTACCTGATGGGAGAGA
40	Ldha-R	GTAGGCACTGTCCACCACCT
41	Tet1F	ACACAGTGGTGCTAATGCAG
42	Tet1R	AGCATGAACGGGAGAATCGG
43	Tet2F	AGAGAAGACAATCGAGAAGTCGG
44	Tet2R	CCTTCCGTACTCCCAAACTCAT
45	Tet3F	TGCGATTGTGTCGAACAAATAGT
46	Tet3R	TCCATACCGATCCTCCATGAG
47	Dnmt1-F	AAGAATGGTGTTGTCTACCGAC
48	Dnmt1-R	CATCCAGGTTGCTCCCCTTG
49	Dnmt3a-F	GAGGGAACTGAGACCCCAC
50	Dnmt3a-R	CTGGAAGGTGAGTCTTGGCA
51	Dnmt3b-F	AGCGGGTATGAGGAGTGCAT
52	Dnmt3b-R	GGGAGCATCCTTCGTGTCTG
53	Methy-cyclinD1-F	GAGTTTGTACGAGAGTTTAGGGTTC
54	Methy-cyclinD1-R	AAAAATAAATACGTTTCCGAATACG
55	Unmethy-cyclinD1-F	GTTTGTATGAGAGTTTAGGGTTTGT
56	Unmethy-cyclinD1-R	AAATAAATACATTTCCAAATACACC

## Abbreviations

3D culture system	three dimensional semisolid culture system
αKG	a-ketoglutarate
5mc	5-methylcytosine
5hmc	5-hydroxymethylcytosine
CK7	cytokeratin
EGF	epidermal growth factor
FBS	fetal bovine serum
Nkx2.2	Nk2 homeobox 2
Nkx6.1	Nk6 homeobox 1
Pax4	paired box 4
Pax6	paired box 6
Pdx1	pancreatic and duodenal homeobox 1
Sox9	(sex determining region Y)-box 9
ROS	Reactive oxide species
MSP	methylation-specific PCR,
Oligo	oligomycin A
2-DG	2-Deoxy-d-glucose
Tet	tet methylcytosine dioxygenase
TCA	Tricaboxylic acid cycle
Cs	citrate synthase
Idh	isocitrate dehydrogenase
Ogdh	oxoglutarate (alpha-ketoglutarate) dehydrogenase
KEGG	Kyoto Encyclopedia of Genes and Genomes

## Appendix A

### High Throughput Sequencing

Three independent samples of colonies and their pre-culture controls isolated from mouse pancreas were harvested for HTS. RNA was extracted as previously mentioned. All following sequencing and data were performed and analyzed by Genergy Inc. (Shanghai, China).

## Appendix B

We employed the 3D medium as the cell culture system (Figure A3a). The cells were seeded in the 3D culture system without any additional treatment for 2 weeks. During this time, several “ring” colonies formed spontaneously in the 3D culture and the colonies became gradually larger (Figure A3b,c). The proportion of the CD133^+^ cell population reached 93.5% (Figure A3d). Self-renewal and proliferation are the two properties of pancreatic progenitors; thus, to assess the self-renewal ability, the ring colonies in the culture medium were all hand-picked and digested into single cells. Subsequently, the single cells were counted and were re-seeded in the same 3D culture system (10,000 cells per well). Two weeks later, secondary ring colonies emerged. Both the colony-forming frequency and the cell number per well were significantly increased relative to the first passage (Figure A3e,f). The cell colony gene expression of several progenitor-related genes—Pdx1, Hnf1b, Sox9 and CD133—and proliferation-related genes—CyclinD1 and Ki67—were all elevated while the expression of differentiation-related genes—ngn3, Pax6, ins1, ins2—were decreased relative to the pancreatic single cells. Moreover, the high expression of CK7 confirmed that the cell colonies are ductal in origin in comparison to the pancreatic single cells (Figure A3g–i). These results highly support the use of the 3D culture system for the enrichment of CD133^+^ cells.

## Appendix C

**Table A1:** Significantly changed genes enriched in different Kyoto Encyclopedia of Genes and Genomes (KEGG) pathways (represented by *p*-values) in progenitor-like cells cultured in vitro compared to pancreatic single cells.

Pathway Name	Significantly Enriched Genes	All Genes of Pathway	*p*-Value
Metabolic pathways	543	1187	3.46 × 10^−32^
Oxidative phosphorylation	84	135	5.07 × 10^−12^
Protein processing in endoplasmic reticulum	92	162	5.33 × 10^−10^
PI3K-Akt signaling pathway	152	334	0.000000555
Aminoacyl-tRNA biosynthesis	28	41	0.00000586
HIF-1 signaling pathway	59	113	0.0000266
Pyruvate metabolism	25	41	0.000297
MAPK signaling pathway	109	252	0.000361
Glycine, serine and threonine metabolism	23	39	0.001
Fatty acid metabolism	26	46	0.00115
Citrate cycle (TCA cycle)	19	31	0.00146
*N*-Glycan biosynthesis	27	49	0.00155
Notch signaling pathway	26	49	0.00379
Cysteine and methionine metabolism	20	36	0.0055
Amino sugar and nucleotide sugar metabolism	25	48	0.00616
Maturity onset diabetes of the young	15	26	0.01
Glutathione metabolism	25	50	0.0118
Fructose and mannose metabolism	20	38	0.0119
Pentose phosphate pathway	16	29	0.0138
Beta alanine metabolism	16	29	0.0138
Insulin signaling pathway	58	137	0.0164
Glyoxylate and dicarboxylate metabolism	14	25	0.0178
Alanine, aspartate and glutamate metabolism	17	32	0.0178
Purine metabolism	67	165	0.0271
Porphyrin and chlorophyll metabolism	21	43	0.0273
Sphingolipid metabolism	20	41	0.0313
Pyrimidine metabolism	41	96	0.0356
D-Glutamine and D-glutamate metabolism	3	3	0.0383
TGF-beta signaling pathway	36	84	0.0451
